# Processing of *Alu* small RNAs by DICER/ADAR1 complexes and their RNAi targets

**DOI:** 10.1261/rna.076745.120

**Published:** 2020-12

**Authors:** Yusuke Shiromoto, Masayuki Sakurai, Helen Qu, Andrew V. Kossenkov, Kazuko Nishikura

**Affiliations:** The Wistar Institute, Philadelphia, Pennsylvania 19104, USA

**Keywords:** RNA editing, ADAR1, DICER, RNAi, *Alu*, siRNA

## Abstract

In addition to adenosine-to-inosine RNA editing activities, ADAR1 has been shown to have various RNA editing-independent activities including modulation of RNAi efficacy. We previously reported that ADAR1 forms a heterodimer complex with DICER and facilitates processing of pre-miRNAs to mature miRNAs. In addition to miRNA synthesis, DICER is involved in processing of long dsRNAs into small RNAs (endo-siRNAs). Generation of retrotransposon-derived endo-siRNAs by DICER and their functions in regulation of transcripts in mouse oocytes has been previously reported. However, the synthesis and functions of endo-siRNAs in somatic cells remain largely unknown. Here, we report that ADAR1 together with DICER generates endogenous small RNAs, *Alu* endo-siRNAs by cleaving long double-stranded regions of inverted *Alu* repeats. We identified AGO2-loaded *Alu* endo-siRNAs, which are highly expressed in commonly used cell lines. These *Alu* endo-siRNAs carrying both sense and antisense *Alu* sequences seem to target a set of genes containing a single *Alu* sequence, either antisense or sense, respectively, within their 3′UTR. In silico screening identified potential RNA silencing target genes for these *Alu* endo-siRNAs. We present results of a proof-of-concept experiment, in which sense *Alu* endo-siRNAs derived from *AluSz* and *AluJr* family elements target CUB Domain Containing Protein 1 mRNAs containing an antisense copy of *AluJb* in their 3′UTRs and consequently induce apoptosis in HeLa cells. Our results clearly indicate that *Alu* endo-siRNAs are functional also in somatic cells.

## INTRODUCTION

RNA editing that converts adenosine to inosine (A-to-I RNA editing) specifically in double-stranded RNAs (dsRNAs) is catalyzed by adenosine deaminases acting on RNA (ADARs) (Mannion et al. 2015; Nishikura 2016; Walkley and Li 2017; Eisenberg and Levanon 2018). Three ADAR gene family members (ADAR1–3) have been identified in vertebrates. Members of the ADAR gene family share common structural features, such as the presence of multiple dsRNA binding domains (dsRBDs) and a separate deaminase domain (Lai et al. 1995; Macbeth et al. 2005). Although both ADAR1 (ADAR) and ADAR2 (ADARB1) are catalytically active enzymes, catalytic activities of ADAR3 (ADARB2) have not been shown yet. As the translation machinery reads inosine as guanosine, A-to-I RNA editing of neurotransmitter and ion channel gene transcripts result in recoding and diversification of their functions (Hood and Emeson 2012). For instance, the physiologically important recoding-type editing target site of ADAR2, the Q/R site of α-amino-3-hydroxy-5-methyl-isoxazole-4-propionate (AMPA) glutamate receptor GluR-2 mRNAs, has been reported (Higuchi et al. 2000; Hood and Emeson 2012). In contrast, a wide range of ADAR1 functions have been reported (Mannion et al. 2015; Nishikura 2016). There are two ADAR1 isoforms, a full-length interferon-inducible ADAR1p150 and a shorter and constitutive ADAR1p110 truncated at the amino terminus. ADAR1p110 mainly localizes in the nucleus, whereas ADAR1p150 is mostly detected in the cytoplasm (Patterson and Samuel 1995). The inactivation of ADAR1 in mice leads to an embryonic lethal phenotype because of widespread apoptosis, revealing that requirement of ADAR1 functions for development (Hartner et al. 2004; Wang et al. 2004). Although ADAR1 edits select protein coding sequences (Song et al. 2016) as well as certain microRNA precursors (Yang et al. 2006; Kawahara et al. 2007; Iizasa et al. 2010), noncoding sequences consisting of inverted repeats of retrotransposon elements such as *Alu* and *SINE* are the most frequent targets of ADAR1 (Bazak et al. 2014; Porath et al. 2014; Sakurai et al. 2014; Tan et al. 2017). Studies by several independent groups revealed that aberrant activation of the dsRNA-sensing mechanism mediated by the MDA5–MAVS–IFN pathway and consequent interferon (IFN) and inflammatory responses underlie the embryonic lethal phenotype of *Adar1* null mice (Mannion et al. 2014; Liddicoat et al. 2015; Pestal et al. 2015). The cytoplasmic ADAR1p150 isoform specifically suppresses this dsRNA-sensing pathway by hyper-editing *SINE* and *Alu* dsRNAs present in 3′UTRs of certain mRNAs (Mannion et al. 2014; Liddicoat et al. 2015; Pestal et al. 2015; Ahmad et al. 2018). Interestingly, a more recent study indicates the presence of currently unknown non-*Alu* long dsRNAs as alternate targets of ADAR1p150, rather than 3′UTR *Alu* dsRNAs (Barak et al. 2020). Regardless of the exact triggers of the dsRNA-sensing mechanism, loss of this particular function of ADAR1 has been linked to the severe autoimmune disease Aicardi–Goutières syndrome (AGS) (Rice et al. 2012; Pestal et al. 2015) and underlies the resistance developed in certain tumors to PD-1 immune checkpoint blockade-based immunotherapy (Ishizuka et al. 2019). These ADAR1 functions described depend on its A-to-I RNA editing activities, which require homodimerization through its dsRBD3 (Cho et al. 2003; Valente and Nishikura 2005; Ota et al. 2013).

Furthermore, several RNA editing-independent functions of ADAR1 have also been reported. The normally nuclear-localized ADAR1p110 moves to the cytoplasm upon phosphorylation by MAP kinases and promotes survival of stressed cells by protecting antiapoptotic gene transcripts from Staufen1-mediated mRNA decay (Sakurai et al. 2017). This stress response function of ADAR1p110 has been shown to be RNA editing-independent (Sakurai et al. 2017). We found that ADAR1 forms a complex with DICER, a member of the RNase III gene family and an essential enzyme involved in miRNA processing and the RNA interference (RNAi) mechanism (Ota et al. 2013). ADAR1 in the complex increases *V*_max_ of the DICER pre-miRNA cleavage reaction and promotes loading of miRNA onto RISC, identifying a new role of ADAR1 in miRNA processing and RNAi mechanisms and revealing a stimulative interaction between RNA editing and RNAi (Nishikura et al. 2013; Ota et al. 2013). The amino-terminus half of the DICER DExD box RNA helicase domain and the ADAR1 dsRBD2 are required for formation of the DICER/ADAR1 heterodimer complex (Ota et al. 2013). This RNAi function of ADAR1 is also RNA editing-independent (Ota et al. 2013). Initial detection of the DICER/ADAR1 complex formation in developing mouse embryos and also in HeLa cells has been recently extended to oral squamous carcinoma cells (Liu et al. 2019a) and mouse cardiomyocytes undergoing viral myocarditis (Zhang et al. 2019). Interestingly, involvement of DICER in synthesis of endo-siRNAs from retrotransposon-related repetitive elements such as *Alu* and 7SL has been reported (Watanabe et al. 2008; Ren et al. 2012). In this study, we investigated in vitro processing of *Alu* dsRNAs to *Alu* endo-siRNAs by DICER/ADAR1 complexes. We found that ADAR1 significantly promotes processing of *Alu* dsRNA to *Alu* siRNAs. Furthermore, we identified various AGO2-bound endogenous *Alu* siRNAs likely to be processed by DICER and ready to act on their target genes in vivo in HEK293T cells. We here report targeting by such *Alu* endo-siRNAs of CUB Domain Containing Protein 1 (CDCP1) mRNAs containing an antisense copy of *AluJb* in their 3′UTRs (Gong and Maquat 2011), and consequent induction of apoptosis, thus demonstrating *Alu* endo-siRNA-mediated gene silencing in somatic cells.

## RESULTS

### No cleavage of *Alu* RNAs by DICER alone or DICER/ADAR1 complexes

**FIGURE 1.**
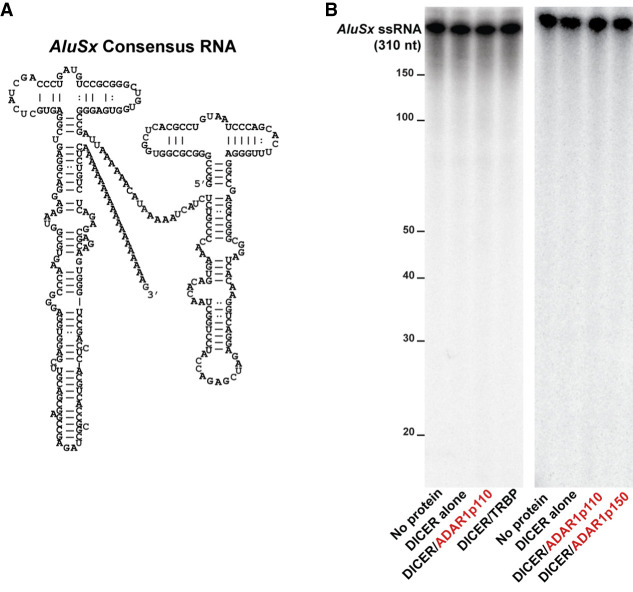
DICER does not cleave single *Alu* RNAs. (*A*) Secondary structure of single *AluSx* RNA. (*B*) The DICER cleavage reaction was done at 37°C for 60 min with 0.15 nM of *AluSx* RNA and 1.5 nM of DICER alone or various DICER complexes.

It has been reported that DICER cleaves cytotoxic *Alu* RNAs, ∼300 nt in length, transcribed by RNA polymerase III (Fig. 1A) and prevents their accumulation and consequent induction of apoptosis in retinal pigmented epithelium (RPE) cells. Deficiency in this particular DICER function has been suggested to underlie age-related macular degeneration (AMD), a leading cause of blindness (Kaneko et al. 2011; Tarallo et al. 2012). However, several studies arguing against this AMD model have also been reported (Sundermeier et al. 2017; Kosmidou et al. 2018). Furthermore, processing of 7SL RNA, also transcribed by RNA polymerase III and considered as an ancestral RNA of *Alu* RNAs, into small RNAs by DICER has been suggested (Ren et al. 2012). To reexamine whether DICER indeed cleaves *Alu* RNAs and also to see whether ADAR1 in any way facilitates such DICER activity, we conducted in vitro assays using DICER/ADAR1 and DICER/TRBP (TARBP2) complexes as well as preparation of highly purified DICER alone and *AluSx* RNAs (302 nt in length). Differentially epitope tagged FLAG–DICER/HAT–ADAR1 and FLAG–DICER/HAT–TRBP complexes were purified by the baculovirus coexpression system and sequential epitope-based affinity chromatography purification as described previously (Ota et al. 2013), whereas a uniformly ^32^P-labeled *Alu* RNA containing a single copy of the sense *AluSx* sequence was prepared by in vitro transcription. We found that a single copy sense *Alu* RNA was resistant to cleavage by DICER alone in contrast to the previous report (Kaneko et al. 2011). Furthermore, DICER/ADAR1p110 and DICER/ADAR1p150 complexes or DICER/TRBP complexes all could not cleave *Alu* RNAs (Fig. 1B). In fact, we detected some degradation of *Alu* RNAs by partially purified DICER protein preparations (data not shown) prior to establishment of our DICER protein purification protocol. Thus, the previously reported in vitro processing of *Alu* and 7SL RNAs into small fragmented RNAs by DICER could be due to contaminating RNases in the DICER preparation. Alternatively, the *Alu* or *7SL* RNAs used for previous in vitro dicing assays might be contaminated by their antisense strand RNAs, which are often generated during in vitro transcription of sense strand RNAs. Synthesis of such antisense strand RNAs would result in the formation of completely complementary double-stranded *Alu* and *7SL* RNAs: perfect substrates for DICER. We conclude that *Alu* RNAs transcribed by RNA polymerase III are not natural substrates of DICER, perhaps because of their rather short stem structures with many mismatched base pairs (Fig. 1A).

### ADAR1 promotes processing of siRNAs from *Alu* dsRNAs by DICER

**FIGURE 2.**
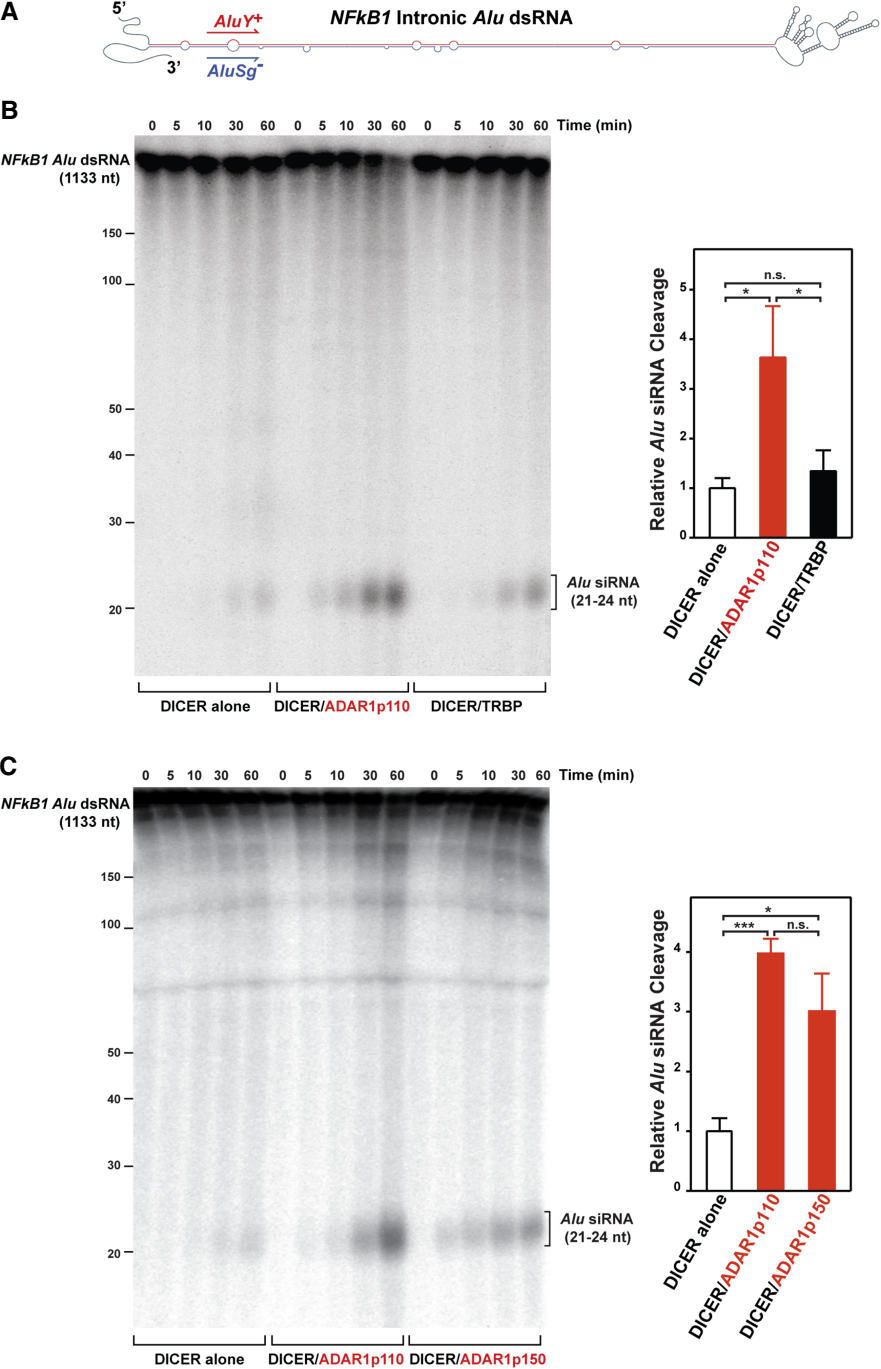
ADAR1 augments the DICER cleavage reaction rate for *NFκB1 Alu* dsRNAs and increases *Alu* siRNA production. (*A*) Secondary structure of *NFκB1* intronic *Alu* dsRNA. (*B*,*C*) The time course analysis of DICER cleavage. The DICER reaction was done at 37°C with 0.15 nM of *NFκB1 Alu* dsRNA and 1.5 nM of DICER alone or DICER complexes for various times. The reaction products were fractionated by 10% Urea-PAGE. The *Alu* siRNA cleavage efficiencies were determined at the 30 min time point. Data are shown as mean ± SD. (*n* = 3 technical replicates). (*) *P* < 0.05; (***) *P* < 0.001; n.s. not significant by two-tailed Student's *t*-test.

In addition to cleavage of pre-miRNAs to mature miRNAs, DICER also cleaves long dsRNAs into siRNAs (Zhang et al. 2002; Lee et al. 2004). We next investigated whether DICER processes *Alu* dsRNA stems of long hairpin-like structures consisting of inverted repeats of sense and antisense *Alu* sequences and various sizes of connecting loop (Fig. 2A). We first tested a uniformly ^32^P-labeled *Alu* dsRNA hairpin structure consisting of a sense *AluY*^*+*^, an antisense *AluSg*^*−*^, and a 533 nt long loop (Fig. 2A), which is embedded within the intron 16 of the *NFκB1* gene (Kawahara and Nishikura 2006). We found that DICER alone cleaved *Alu* dsRNA into 21–24 nt long small RNAs (*Alu* siRNAs). Most interestingly, both ADAR1p110 and ADAR1p150 as DICER/ADAR1 complexes promoted DICER cleavage of *Alu* dsRNA to *Alu* siRNAs by approximately three- to fourfold (Fig. 2B,C). In contrast, TRBP had very little effect on the DICER activity of *Alu* dsRNA cleavage (Fig. 2B). We also tested an additional *Alu* dsRNA hairpin structure consisting of a sense *AluSp*^+^, an antisense *AluSp*^*−*^, and a 70 nt long loop (Supplemental Fig. S1A), which is in the 3′UTR of *NICN1* gene (Chen et al. 2008). Complementarity of the *NICN1 Alu* dsRNA stem formed by sense and antisense of the same *Alu* element subfamily (*AluSp*) is much higher than that of the *NFκB1 Alu* dsRNA, and the loop size of the *NICN1 Alu* hairpin structure is much smaller than that of the *NFκB1 Alu* hairpin. In spite of these differences, we obtained similar results: The DICER/ADAR1 complex cleaved *Alu* dsRNA more efficiently and generated more *Alu* siRNAs than DICER alone (Supplemental Fig. S1B). ADAR1 promotion of DICER activities to cleave pre-miRNAs to mature miRNAs has been reported to be independent of ADAR1 A-to-I editing activities (Ota et al. 2013). Generation of *Alu* siRNAs by the DICER complex with an ADAR1p110–E912A editing defective mutant was as effective as that of the DICER/ADAR1p110–WT complex (Supplemental Fig. S1C), indicating that ADAR1 promotion of DICER cleavage of *Alu* dsRNA is also independent of the A-to-I editing activities of ADAR1.

*Alu* siRNAs (∼21–24 nt) generated by cleavage of the *NFκB1* *Alu* dsRNA by the DICER/ADAR1p110 complex (Fig. 2C, 60 min time point) were gel-purified and subjected to high-throughput sequencing (small RNA-seq) (Supplemental Fig. S2). Although we found that small RNAs were generated from both sense and antisense strands of the *Alu* hairpin stem region, their read numbers and patterns were not those expected from symmetrical processive cleavage of the completely matched dsRNA (Supplemental Fig. S2). We noticed no small RNA reads corresponding to certain regions containing internal loops and bulges. These results may indicate that DICER/ADAR1 complexes bind *Alu* dsRNA stems all along their length, not merely at the ends, and cleave them wherever bound, instead of acting processively in one direction.

### A-to-I edited *Alu* dsRNAs are also cleaved by the DICER/ADAR1 complex

The most frequent ADAR1 target for A-to-I editing is *Alu* dsRNA (Porath et al. 2014; Sakurai et al. 2014; Tan et al. 2017), and editing may affect their cleavage by DICER. Typical *Alu* hairpin stems contain both matched A:U and mismatched A–C base pairs (Fig. 2A; Supplemental Fig. S1A). In addition to adenosines of matched A:U base pairs, adenosines of mismatched A–C base pairs are known to be edited by ADAR1. ADAR1-mediated editing of adenosine residues of A:U matched base pairs results in I:U wobble base pairs, which decreases double-strandedness of *Alu* hairpin stems. On the other hand, editing of A–C mismatched base pairs results in I:C matched base pairs, which increases double-strandedness. Therefore, we next examined the effects of A-to-I editing on DICER cleavage of *Alu* dsRNAs. We first edited in vitro *NFκB1* and *NICN1 Alu* dsRNA hairpins as much as possible using purified FLAG-ADAR1p110 proteins (Supplemental Fig. S3A) prior to in vitro dicing assay. Interestingly, we found very little effects of A-to-I editing: Both unedited and edited *Alu* dsRNAs were cleaved by the DICER/ADAR1p110 complex to siRNAs equally well (Supplemental Fig. S3B). Possibly due to the offsetting effects of A:U editing and A–C editing, A-to-I editing does not change significantly the overall double-strandedness of most *Alu* hairpins and consequently has very little effects on their DICER cleavage, in contrast to the reported antagonistic interaction of A-to-I editing and RNAi on long dsRNAs in *C. elegans* (Wu et al. 2011).

### In vivo identification of *Alu* siRNAs and their targets in commonly used cell lines

**FIGURE 3.**
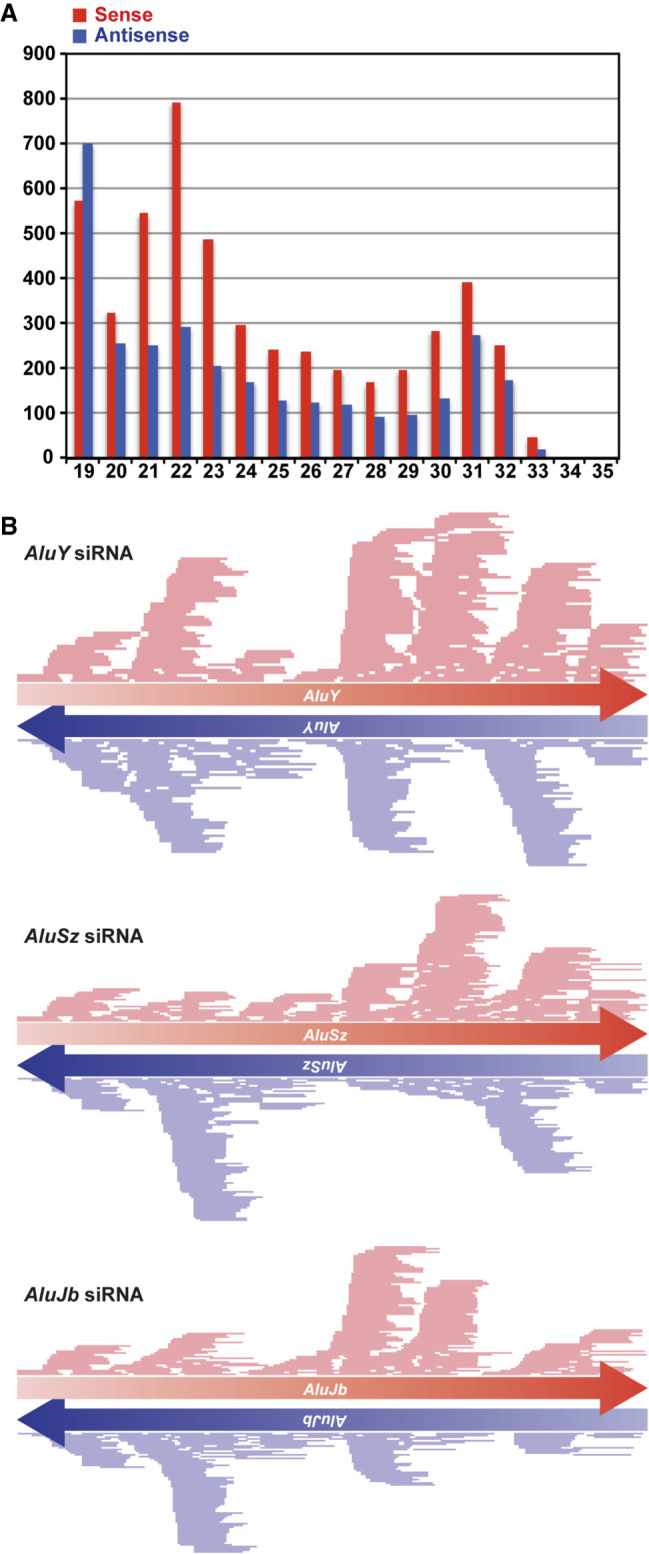
*Alu* siRNA expression in HeLa cells. (*A*) The length distribution of *Alu*-derived small RNAs in HeLa cells. *y*-axis: counts per million reads; *x*-axis: base length. (*B*) Distribution of *Alu* siRNAs corresponding to sense and antisense strands of consensus *AluY*, *AluSz*, and *AluJb* sequences (UCSC genome database). RNAs of 19 to 24 nt in length were used as siRNAs.

**FIGURE 4.**
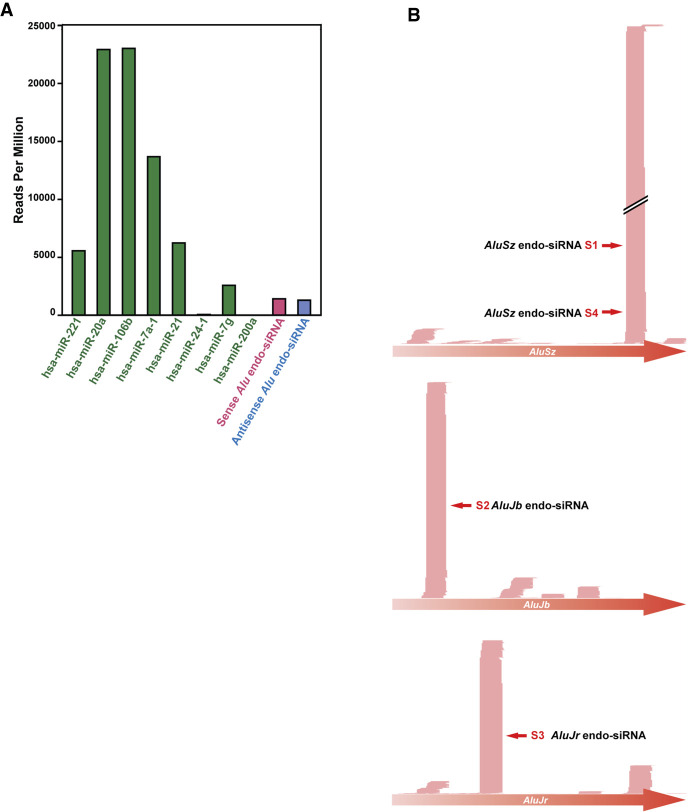
*Alu* siRNAs are loaded onto AGO2. (*A*) The read counts per million reads of various mature miRNAs and *Alu* siRNAs in small RNAs immunoprecipitated with AGO2 antibodies (GSM1334330). (*B*) Distribution of AGO2-bound *Alu* siRNAs corresponding to sense strands of *AluSz* (chr2:5532106–5532415), *AluJb* (chr1:236404589–236404810), and *AluJr* (chr1:227459304–227459613).

We previously investigated expression of miRNAs in HeLa cells by small RNA-seq analysis (GSM1057798) (Ota et al. 2013). We reexamined our small RNA-seq data and found that both sense and antisense strands of *Alu*-endo siRNAs ranging from 19 to 33 nt with a peak ranging from 21 to 24 nt in length can be detected in HeLa cells (Fig. 3A), although their expression levels are much lower than those of miRNAs: *Alu* siRNAs accounted for only 0.3% of total miRNAs. In vivo detection of *Alu* siRNAs at a slightly higher level (1.2% of total miRNAs) has been also reported in HepG2.2.15 cells (Ren et al. 2012). We annotated HeLa *Alu* siRNAs to various *Alu* subfamily consensus sequences, which resulted in sense and antisense strand read mapping patterns expected for DICER cleavage products, as shown for three examples: *AluY*, *AluSz*, and *AluJb* subfamilies (Fig. 3B). In addition, profiling of AGO2-bound small RNAs has been reported in HEK293T cells (GSM1334330) (Rybak-Wolf et al. 2014). These AGO2-bound small RNAs are considered as productive DICER cleavage products ready to be engaged in RNAi (Rybak-Wolf et al. 2014). We examined those published small RNA-seq data and confirmed that AGO2-bound sense and antisense *Alu* endo-siRNAs can be detected in HEK293T cells (Supplemental Table S3), although their expression levels were much lower than those of AGO2-bound miRNAs (only 0.09% of AGO2-bound miRNAs) (Fig. 4A). These results indicate that *Alu* endo-siRNAs are produced and also that some of them may be functional, targeting cellular genes via RNA interference in commonly used somatic cell lines such as HeLa and HEK293T.

**FIGURE 5.**
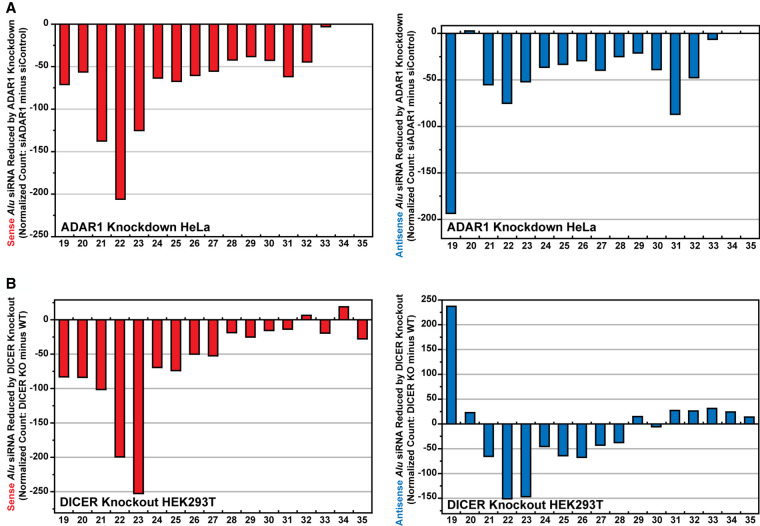
Reduced expression of *Alu* siRNAs in ADAR1 or DICER depleted cells. (*A*) The length distribution of read numbers of sense and antisense *Alu* siRNAs reduced by ADAR1 knockdown in HeLa cells. (*B*) The length distribution of read numbers of sense and antisense *Alu* siRNAs reduced by DICER knockout in HEK293T cells. Read numbers were first normalized: The read number of *Alu* siRNAs was divided by the read number excluding miRNA and *Alu* siRNAs from the total small RNA read number and multiplied by one million. Then, the normalized *Alu* siRNA read number of ADAR1 knockdown (siADAR1 HeLa) or DICER knockout (*DICER* null HEK293T) cells was subtracted from that of control cells (siControl HeLa or WT HEK293T), and the difference was plotted on the *y*-axis. A negative number on the *y*-axis indicates a decrease in the normalized number of reads after ADAR1 knockdown or DICER knockout. The *x*-axis is the number of bases in the siRNA sequence.

To evaluate the in vivo effects of ADAR1 and DICER on synthesis of these *Alu* siRNAs, we examined *Alu* siRNA reads in ADAR1 knockdown HeLa cells (GSM105779) (Ota et al. 2013) in comparison to control cells. We found that both sense and antisense *Alu* siRNA read counts, peaking at 21–23 (major peak) and 30–32 (minor peak) nucleotides in length, are reduced significantly by ADAR1 depletion (Fig. 5A). As expected, DICER depletion resulted also in significant reduction of both sense and antisense *Alu* siRNA reads, peaking at 21–23 nt in length, in DICER knockout HEK293T cells (Fig. 5B; Bogerd et al. 2014). These results confirmed the in vivo contributions of ADAR1 and DICER to genesis of *Alu* endo-siRNAs in somatic cell lines.

Annotation of AGO2-bound sense and antisense *Alu* siRNAs identified in HEK293T cells (Fig. 4A) to known *Alu* subfamily sequences revealed the presence of several high read *Alu* siRNAs originating from certain *Alu* subfamily members such as *AluSz*, *AluJb*, and *AluJr* for sense *Alu* siRNAs (Fig. 4B) and *AluSp*, *AluYj4*, *AluYf1*, and *AluSx* for antisense *Alu* siRNAs (Supplemental Fig. S4). Whereas sense *Alu* siRNAs seemed to be generated from several separate regions of each *Alu* subfamily sequence (Fig. 4B), antisense *Alu* siRNAs appeared to originate from a select region of the antisense strand *Alu* sequences (Supplemental Fig. S4), possibly indicating a preference of AGO2 binding to *Alu* siRNAs carrying a specific sequence.

### Sense *Alu* siRNAs target CDCP1 and induce apoptosis in HeLa cells

**FIGURE 6.**
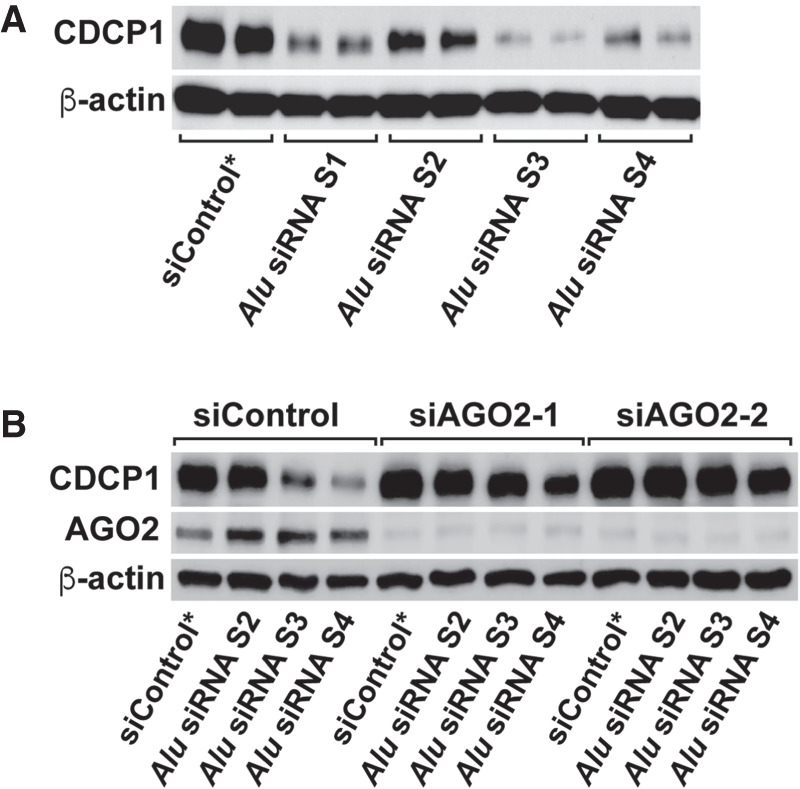
*Alu* siRNAs target CDCP1 in an AGO2-dependent manner. (*A*) Western blotting analysis of CDCP1 (full-length 135 kDa bands) in *Alu* siRNA-transfected HeLa cells at 72 h post-transfection. β-actin was used as a loading control. (*B*) The CDCP1 levels in *Alu* siRNA-transfected AGO2 knockdown cells were evaluated by western blotting. HeLa cells were treated with AGO2 siRNA twice. *Alu* siRNA and the second AGO2 siRNA were transfected into HeLa cells 48 h after the first AGO2 siRNA treatment.

**FIGURE 7.**
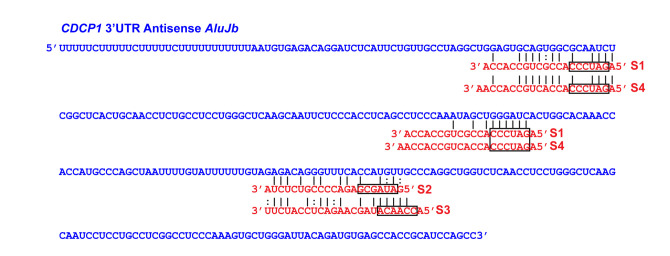
Predicted binding sites of *Alu* siRNAs in the 3′UTR of *CDCP1* mRNAs. One putative target site of *Alu* siRNA-S2 or -S3 is located in the antisense *AluJb* located in the *CDCP1* 3′UTR. Two potential binding sites of *Alu* siRNA-S1 or -S4 are located in the *CDCP1* 3′UTR. Black box: seed sequence. The *AluJb* in the *CDCP1* 3′UTR located at chr3:45083628–45083942. S2 and S3 *Alu* siRNAs most likely originated from their target site regions of the corresponding sense *Alu* strand. Similarly, S1 and S4 *Alu* siRNAs likely originated from their first (upstream) target site regions of the corresponding sense *Alu* strand.

**FIGURE 8.**
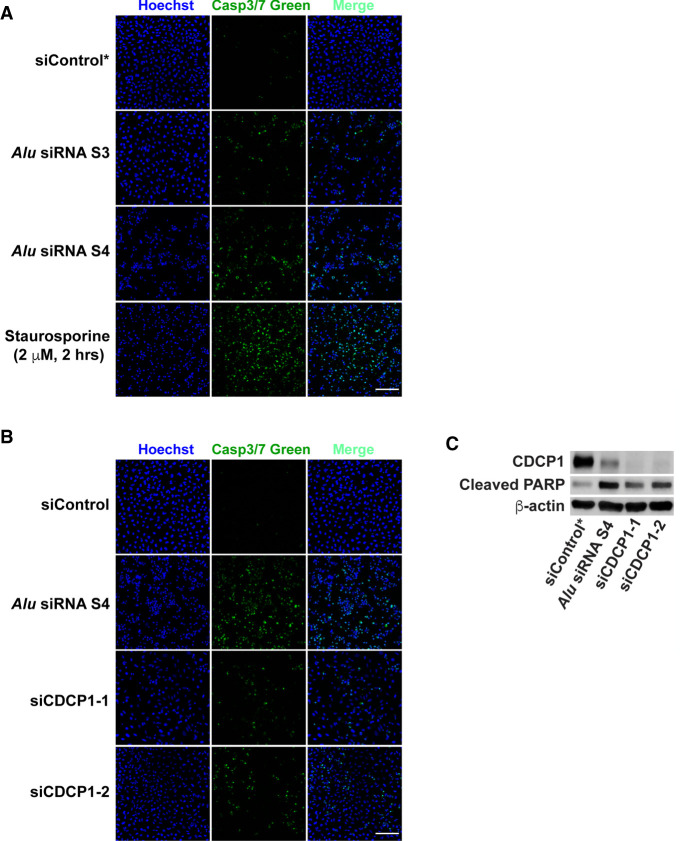
*Alu* siRNAs induce apoptosis. (*A*) Fluorescence microscopic detection of apoptosis induced in *Alu* siRNA-transfected HeLa cells in comparison to the cells treated with a known inducer of apoptosis, Staurosporine (2 µM, 2 h). Apoptosis was evaluated with the CellEvent Caspase-3/7 Green apoptosis-detection system (Life Technologies). (*B*) Apoptosis induced in CDCP1 knockdown HeLa cells was also evaluated using the CellEvent Caspase-3/7 Green apoptosis-detection system. Scale bars, 200 µm. (*C*) The extent of apoptosis induced by *Alu* siRNA transfection or CDCP1 knockdown was analyzed by western blotting of cleaved poly(ADP-ribose) polymerase (PARP). CDCP1 bands represent the full-length protein (135 kDa), whereas PARP bands represent the cleaved protein (89 kDa).

Although binding to AGO2 (Rybak-Wolf et al. 2014) and high expression levels of certain *Alu* endo-siRNAs (Supplemental Table S3) indicate that they could certainly be engaged in RNA interference-mediated silencing of target genes, it is not known whether they are indeed functional in somatic cell lines. To obtain evidence for functionality of *Alu* endo-siRNAs in vivo, we chose four sense *Alu* siRNAs, S1 (*AluSz*), S2 (*AluJb*), S3 (*AluJr*), and S4 (*AluSz*) (Fig. 4B) and looked for their potential target genes containing a single copy of the antisense *Alu* in their 3′UTR regions. Using the UCSC Human Genome Database and HeLa App (Liu et al. 2019b), we first identified 155 genes, which are expressed at relatively high levels and contain a single copy of 3′UTR antisense *Alu* sequences in HeLa cells (Supplemental Table S4A). Similarly, we also identified 165 genes containing a single copy of 3′UTR sense *Alu* sequences (Supplemental Table S4B), which could be potential targets of antisense *Alu* siRNA, AS1–AS4 (Supplemental Fig. S4). Among the candidate genes containing a single copy of 3′UTR antisense *Alu* sequences, we further selected the genes known to regulate apoptosis, which can be easily determined as a phenotype of gene silencing (Supplemental Table S4A). To this end, we picked CUB Domain Containing Protein 1 (CDCP1) containing a single antisense copy of *AluJb* subfamily sequence within its 3′UTR region (Scherl-Mostageer et al. 2001) as a test target gene for sense *Alu* siRNAs, S1–S4 (Supplemental Table S4A, highlighted in yellow). CDCP1, a transmembrane protein overexpressed in many types of cancers, suppresses apoptosis and thereby promotes their metastasis (Uekita et al. 2007; Deryugina et al. 2009). We transfected separately four different sense *Alu* siRNAs (S1–S4) into HeLa cells. Their silencing effects were determined by western blotting analysis, which revealed that all four sense *Alu* siRNAs indeed suppressed expression of CDCP1. However, their silencing effects varied: S1, S3, and S4 were very effective, whereas S2 was least effective (Fig. 6A). The difference is likely a result of differences in their seed sequence complementarity to target site sequences within the 3′UTR antisense *AluJb* of CDCP1 mRNAs (Fig. 7). Furthermore, we found that S1 and S4 *Alu* siRNAs are more effective because they have two target sites, especially the second site with perfect complementarity between their seed sequences and target sequences (Fig. 7). Their silencing effects were completely dependent on AGO2, since depletion of AGO2 abolished their silencing effects on the expression of CDCP1 (Fig. 6B). As expected by its already known antiapoptotic function, suppression of CDCP1 by *Alu* siRNA S3 and S4 (Fig. 8A) as well as two separate siCDCP1 RNAs targeting the CDCP1 coding region (Fig. 8B) resulted in induction of significant apoptosis, determined by fluorescence microscopy detection of activated Caspase-3/7 (Fig. 8A,B) and also by western blotting analysis for cleaved PARP fragments (Fig. 8C). These results certainly proved that *Alu* siRNAs are functional and capable of silencing their target gene via AGO2-dependent RNAi mechanism.

## DISCUSSION

It has been reported that there are 333 human genes that contain inverted *Alu* repeats within their 3′UTRs (Chen et al. 2008). These 3′UTR *Alu* dsRNAs, if not edited by the cytoplasmic ADAR1p150, activate the MDA5–MAVS–IFN signaling pathway (Mannion et al. 2014; Liddicoat et al. 2015; Pestal et al. 2015). Furthermore, loss of this particular ADAR1 function in suppression of MDA5–MAVS–IFN signaling underlies AGS pathogenesis caused by ADAR1 mutations (Rice et al. 2012; Mannion et al. 2014) and also the resistance of tumors to immune checkpoint blockade (Ishizuka et al. 2019). In this study, we showed that the long stem regions of *Alu* dsRNAs, despite their imperfect complementarity, can be cleaved by DICER, generating 21–24 nt length *Alu* siRNAs. Furthermore, we found that ADAR1 promotes this DICER activity for processing of *Alu* siRNAs by forming the DICER/ADAR1 complex, as we previously reported for processing of pre-miRNAs to mature miRNAs (Ota et al. 2013). Interestingly, TRBP had almost no DICER-promoting effects for processing of *Alu* dsRNAs to *Alu* siRNAs. The exact mechanism for how ADAR1 promotes DICER activities to process *Alu* dsRNAs to *Alu* siRNAs is currently not known. However, we previously reported that ADAR1 forms a heterodimer complex with DICER via its second dsRNA binding domain and the amino-terminal DExD helicase domain of DICER (Ota et al. 2013). Autoinhibitory function of this DExD helicase domain in the DICER activity for processing of long dsRNAs but not miRNA precursors has been reported: Deletion of DExD helicase domain increased dicing rate by eightfold (Ma et al. 2008). It is believed that the helicase domain affects the overall folding and prevents formation of the most effective structure of DICER (Ma et al. 2008). In fact, a Dicer isoform (Dicer^o^) lacking the amino-terminal DExD helicase domain, expressed only in oocytes, is highly active in processing endo-siRNAs from long dsRNAs (Flemr et al. 2013). Taken together, the interaction of ADAR1 with the DICER DExD helicase domain likely masks its autoinhibitory function, thereby allowing DICER to process *Alu* dsRNAs more efficiently into *Alu* siRNAs.

Expression of endo-siRNAs processed by DICER from long dsRNAs consisting of repetitive sequences of retrotransposons and pseudogene transcripts has been reported in mouse oocytes (Tam et al. 2008; Watanabe et al. 2008; Flemr et al. 2013). Furthermore, function of endo-siRNAs in RNAi-mediated regulation of oocyte transcripts and its essential role in meiosis have been indicated (Murchison et al. 2007; Liu et al. 2010; Flemr et al. 2013). For instance, oocyte gene transcripts containing 3′UTR *SINEs* seem to be silenced by *SINE* siRNAs processed by DICER as a part of the mechanism to regulate retrotransposon activities (Murchison et al. 2007). Interestingly, detection of ADAR1p110 at high levels in oocytes, zygotes, and very early embryos (2–4 cell stages) but rapid elimination at preimplantation stages have been reported with mouse (Garcia-Lopez et al. 2013) and also with human (Qiu et al. 2016). Intriguingly, the DICER expression pattern in maturing oocytes is very similar to that of ADAR1: intense expression in germinal vesicles and rapid decrease at 2–4 cell stage embryos (Murchison et al. 2007). A significant increase in the levels of transcripts derived from retrotransposons is detected in *Dicer null* oocytes due to deficiency in generation of endo-siRNAs (Murchison et al. 2007; Flemr et al. 2013). One possible function of ADAR1 in oocytes may be to promote the DICER activity for processing of endo-siRNAs from repetitive elements such as *Alu* and thereby regulate retrotransposon activities in female germ cells. Deficiency in this regulation appears to underlie failure of meiosis resulting from defective spindle formation and chromosome congression detected in *Dicer null* mouse oocytes (Murchison et al. 2007).

By analyzing small RNA databases, we found that *Alu* siRNAs, although at low levels, are expressed in commonly used somatic cell lines such as HeLa and HEK293T. Furthermore, we identified several highly expressed AGO2-bound sense and antisense *Alu* siRNA, which are likely to be processed by DICER from *Alu* dsRNAs consisting of specific *Alu* subfamily member sequences. We demonstrated, as our proof-of-concept experiment, that these *Alu* siRNAs can be actively engaged in RNAi-mediated silencing of a select target gene *CDCP1*: Suppression of CDCP1 resulted in induction of apoptosis in HeLa cells. CDCP1 has been shown to promote survival of several different types of cancer cells by activating *MYC*, *AKT*, and *Wnt* gene pathways (Majem et al. 2019). Interestingly, infraexpression of *CDCP1* targeting miRNAs miR-654 and miR-218 in ovarian and lung cancers, respectively, has been reported (Chiu et al. 2015; Majem et al. 2019). Furthermore, using miR-654 as a therapeutic to target *CDCP1* and treat ovarian cancers has been proposed (Majem et al. 2019). Our results indicate that *Alu* siRNAs, very effective *CDCP1* targeting small non-coding RNAs, can also be used for treatment of certain cancers.

Although differential expression of ADAR1p150 in the cytoplasm and ADAR1p110 in the nucleus has been previously reported (Patterson and Samuel 1995), they can be detected also in the reciprocal locations in certain somatic cell lines such as HeLa and HEK293T. Furthermore, we previously reported the nuclear export of ADAR1p110 to the cytoplasm regulated by MAP kinases during stress response (Sakurai et al. 2017). Although DICER is thought to be engaged in processing of pre-miRNAs in the cytoplasm, import of DICER phosphorylated in response to DNA damage to the nucleus has been also reported (Burger et al. 2017). Accordingly, it is currently not clear where processing of *Alu* siRNAs takes place within the cell, nor the relative contribution of ADAR1p110 versus ADAR1p150 as the DICER partner in vivo. These issues also muddle the source of *Alu* dsRNAs to be processed to *Alu* siRNAs: nuclear localized intronic *Alu* dsRNAs versus cytoplasmic *Alu* dsRNAs of mRNA 3′UTRs. These issues remain to be addressed in future studies.

In differentiated somatic cells, long dsRNAs induce the interferon (IFN) response via the MDA5-mediated dsRNA-sensing mechanism in contrast to in oocytes and embryonic stem cells where they enter into the RNA interference pathway and are processed to siRNAs by DICER. ADAR1 may contribute to control the potential of long dsRNAs for induction of the MDA5–MAVS–IFN pathway by two ways: introducing extensive A-to-I editing into dsRNAs and thereby suppress MDA5 binding in differentiated somatic cells or promoting the DICER activity and processing dsRNAs to siRNAs in oocytes and ES cells where the IFN pathway is absent. Interestingly, elimination of the IFN pathway components such as MAVS restored the dsRNA-mediated RNA interference pathway in mouse somatic cells, indicating that the dsRNA RNA interference pathway is present also in somatic cells but dominated by the IFN response pathway (Maillard et al. 2016). In this study, we demonstrated that *Alu* siRNAs most likely processed by DICER are generated even in somatic cells such as HeLa and HEK293T, although at very low levels. It has been recently shown that many tumors elevate ADAR1 expression levels and consequently suppress activation of the dsRNA-sensing mechanism mediated by the MDA5–MAVS–IFN pathway, which in turn makes them resistant to immune checkpoint blockade (Ishizuka et al. 2019). It is interesting to know whether ADAR1 also contributes to suppression of IFN responses and development of resistance to immune surveillance in certain tumors by facilitating degradation, rather than by A-to-I editing, of long dsRNAs.

## MATERIALS AND METHODS

### Plasmid construction

The pFastBac-DICER-FLAG plasmid used for recombinant protein purification was prepared by PCR cloning using a FLAG–DICER plasmid (Chendrimada et al. 2005). PCR primers used are listed in Supplemental Table S1. Preparation of baculovirus constructs for HAT–ADAR1p110–WT, HA–ADAR1p110–E912A, and His–ADAR1p150–WT was described previously (Lai et al. 1995; Cho et al. 2003). The *AluSx* sequence was synthesized by Gibson assembly and cloned into pBlueScript II KS (+). Oligonucleotides used for construction of various plasmids are listed in Supplemental Table S1. pBS-NFkB-dsAlu plasmid contains a 1457 bp fragment of an inverted *Alu* repeat of *NFκB* intron. The inverted *Alu* repeat located at chr1:11102837–11267747. The DNA fragment was PCR-amplified using human genomic DNA and PCR primers Bam-NFKBFW2 and Xho-NFKBDW2. Bam-NFKBFW2 contained a BamHI recognition site, and Xho-NFKBDW2 contained a XhoI recognition site. The PCR products were digested with BamHI and XhoI, then inserted into pBluescript II KS (−) vector (Kawahara and Nishikura 2006). pcDNA3.2-NICN1-dsAlu plasmid contained an 895 bp fragment of an inverted *Alu* repeat of *NICN1* 3′UTR. The inverted *Alu* repeat in *NICN1* 3′-UTR located at chr3:49422946–49429326. The DNA fragment was PCR-amplified using human genomic DNA and PCR primers NICN1 1stPCR and NICN1 2ndPCR. S1 tag DNA fragment was synthesized by PCR amplification. The PCR products were digested with SalI and inserted into pENTR/D-TOPO, and then transferred to pcDNA3.2/V5/GW/CAT vector by Gateway recombination.

### Protein purification

The recombinant proteins were purified with a TALON metal resin (Clontech) and/or an anti-FLAG M2 affinity or an anti-HA antibody bead (SIGMA) as described previously (Ota et al. 2013).

### In vitro transcription

Uniformly ^32^P-labeled *Alu* hairpin dsRNA substrates consisting of a partially double-stranded stem made from inverted sense and antisense *Alu* sequences and a loop and a single-stranded *Alu* RNA substrate were prepared by in vitro transcription as described previously (Kawahara and Nishikura 2006). pBS-ssAlu, pBS-NFkB-dsAlu or pcDNA3.2-NICN1-dsAlu were linearized with SwaI, XhoI, or XbaI, respectively, and then transcribed by T7 polymerase (Promega) in the presence of ^32^P-UTP or -ATP at 37°C for 120 min (Dabiri et al. 1996). After DNase I (Promega) treatment, RNAs were purified by denaturing gel electrophoresis and dissolved in annealing buffer containing 10 mM Tris HCl (pH 7.6), 50 mM NaCl. RNAs were incubated at 80°C for 5 min and slowly cooled to room temperature.

### DICER cleavage assay

In vitro DICER cleavage assays were done in a 45 µL reaction mixture containing 20 mM PIPES (pH 6.2), 1.5 mM MgCl_2_, 80 mM NaCl, 1 mM dithiothreitol (DTT), 0.05% NP40, 10% glycerol, 3U/µL RNasin plus inhibitor (Promega), 10 mM EGTA, 1.5 nM DICER, and 0.15 nM substrate RNA. Reaction mixtures were incubated at 37°C, and 7.5 µL aliquots were taken after 0, 5, 10, 30, and 60 min. The reactions were stopped by adding an equal volume of gel loading buffer (80% formamide, 20% glycerol, 0.025% BPB). After heating at 80°C for 10 min, samples were analyzed by 10% Urea-PAGE and quantified using Molecular Dynamics and ImageJ (Ota et al. 2013).

### Analysis of dsRNA structure

Secondary dsRNA structure was calculated using Mfold (Zuker 2003).

### Bioinformatics analysis of small RNA-seq data

We analyzed GSM1057798 (total small RNAs from siControl HeLa cell), GSM1057799 (total small RNAs from siADAR1 HeLa cell), GSM1370372 (total small RNAs from HEK293T cell), GSM1370373 (total small RNAs from DICER knockout HEK293T cell), and GSM1334330 (AGO2-loaded small RNAs from HEK293T cell). We followed a multistep approach to align and annotate small RNA reads as follows: (i) removal of adaptor nucleotides; (ii) removal of siRNA sequences; (iii) alignment to ribosomal RNA sequences; (iv) alignment to transfer RNA sequences; (v) alignment to miRNA sequences; (vi) alignment to repetitive sequences; (vii) alignment to snoRNA sequences; (viii) alignment to RefSeq sequences; and (ix) alignment to human genome sequence. Alignment was performed using the Bowtie program allowing a maximum of two mismatches. One read reported one valid alignment. Unaligned reads were mapped to the next reference sequences. Bioinformatics analysis was performed on the Galaxy platform (Afgan et al. 2018).

More specifically: (i) Removal of adaptor: The 3′ adaptor sequences (GSM1057798, GSM1057799, GSM1370372, and GSM1370373: 5′-TGGAATTCTCGGGTGCCAAGGAACTCCAGTCAC-3′, GSM1334330: 5′-TCTCGTATCGTATGCCGTCTTCTGCTTG-3′) were first removed from the sequences. Trimmed reads of length 19 to 35 nt were subjected to further analysis. (ii) Removal of exogenous siRNA sequences: The reads containing the control siRNA and ADAR1 siRNA sequences used were removed. Reads with 5′-CGTACGCGGAATACTTCGAAG-3′, 5′-TCGAAGTATTCCGCGTACGAT-3′, 5′-CCGCCATCATTATGAAAAAAG-3′ and 5′-TTTTTCATAATGATGGCGGAT-3′ sequences were removed. (iii) Alignment to ribosomal RNAs: The human ribosomal RNA sequences were downloaded from Silva (https://www.arb-silva.de) (Pruesse et al. 2007). (iv) Alignment to transfer RNAs: The human transfer RNA sequences were downloaded from GtRNAdb (http://gtrnadb.ucsc.edu/index.html) (Chan and Lowe 2009). (v) Alignment to miRNA: The human miRNA sequences were downloaded from miRBase (http://www.mirbase.org/ftp.shtml) (Griffiths-Jones et al. 2006). (vi) Alignment to repetitive sequences: The human repetitive sequences (rmsk file) were downloaded from the UCSC genome browser (https://hgdownload.soe.ucsc.edu/downloads.html). The Rmsk file contains *Alu* subfamily sequences. (vii) Alignment to snoRNAs: The human snoRNA sequences were downloaded from snoRNABase (https://www-snorna.biotoul.fr/index.php) (Lestrade and Weber 2006). (viii) Alignment to RefSeq sequences: The human RefSeq sequences were downloaded from the UCSC genome browser (https://hgdownload.soe.ucsc.edu/downloads.html). (ix) Alignment to human genome: The unaligned reads from the previous step were finally aligned to the hg38 human genome.

The alignment data were visualized by the Integrative Genomics Viewer.

### Selection of genes containing a single *Alu* sequence within their 3′UTR

Coding genes with known 3′UTR regions were tested for *Alu* sequences and genes with exactly one *Alu* repeat in the forward orientation (sense) to the gene or exactly one *Alu* in reverse orientation (antisense) to the gene were reported. Highly expressed genes in HeLa cells (CCL2) were picked using HeLa App (proteomics: >log_10_ 4.5). The presence of articles related to apoptosis was examined using a keyword search of the abstract in PubMed.

### Cell culture, small RNA transfection

HeLa cells were cultured in Dulbecco's modified Eagle's medium (DMEM) supplemented with 10% fetal bovine serum (GEMINI) and penicillin/streptomycin at 37°C in 5% CO_2_. HeLa cells were free from mycoplasma contamination. Small-RNA transfection experiments were performed with Lipofectamine RNAiMax (Life Technologies) with a 2 nM final RNA concentration. All small-RNAs used in this study are listed in Supplemental Table S1. Silencer Select siRNAs for human CDCP1(s35060 and s35061) and Negative Control siRNA (s4390844) were purchased from Life Technologies (Supplemental Table S1).

### Western blotting analysis and antibodies used

Cell lysates were prepared in Laemmli buffer (Boston BioProducts) containing benzonase nuclease (Sigma), Complete EDTA-free protease-inhibitor cocktail (Roche), and PhosStop phosphatase-inhibitor cocktail (Roche), and fractionated by 4%–15% SDS–PAGE. Proteins were transferred to Immobilon-P nylon membranes (Millipore). Membranes were blocked with 1% Blocker BSA (Life Technologies) and incubated with primary antibodies (Supplemental Table S2) overnight at 4°C. After incubation with secondary antibodies, membranes were developed with ECL (GE Healthcare).

### In vitro RNA editing

The A-to-I RNA editing reaction of ADAR1p110 was described previously (Wagner et al. 1989).

### Small RNA-seq analysis of in vitro dicing products

In vitro DICER cleavage assays for sequencing were done in a 100 µL reaction mixture containing 20 mM PIPES (pH 6.2), 1.5 mM MgCl_2_, 80 mM NaCl, 1 mM dithiothreitol (DTT), 0.05% NP40, 10% glycerol, 3 U/mL RNasin plus (Promega), 10 mM EGTA, 1.5 nM DICER/ADAR1p110-WT complexes, and 3 nM *NFκB1* ds*Alu* RNA. DICER cleavage products were purified from 10% acrylamide denaturing gel and ligated with 5′ and 3′ adaptors. 3′ cloning adaptors were ligated to the cleavage products using T4 RNA ligase 2-truncated K227Q in a reaction buffer containing NEB ligation buffer, 15% PEG8000, and 1U/µL RNasin plus inhibitor. After denaturing gel purification, 5′ cloning adaptors were ligated to the purified RNAs using T4 RNA ligase1 in a reaction buffer containing NEB ligation buffer, 20% PEG8000, 1U/µL RNasin plus inhibitor, 20 µM ATP, and 10% DMSO. RNA ligation reactions were incubated at 22°C for 120 min. After denaturing gel purification, the ligated RNAs were reverse-transcribed using the RT-primer and SuperScript III (Life Technologies). The reverse-transcribed products were amplified using first and second PCR primers and AccuPrime high fidelity Taq DNA polymerase (Life Technologies). After acrylamide gel purification, PCR products were sequenced by Ion PGM (316 chip, 400 bp) (Life Technologies). Bioinformatics analysis: The 5′ and 3′ adaptor sequences (5′-TGGAATTCTCGGGCACCAAGGT-3′, 5′-ACGCTGGAATTCGCGGTTAAA-3′) were removed from the sequences. Trimmed reads were then mapped to *NFκB1* intronic inverted *Alu* sequence. Alignment was performed using the Bowtie program without any mismatch. Bioinformatics analysis was performed on the Galaxy platform (Afgan et al. 2018).

### Apoptosis analysis: Caspase-3/7 activity and cleaved PARP detection

Apoptotic cells were analyzed by fluorescence microscopy using CellEvent Caspase-3/7 Green apoptosis-detection system (Life Technologies), which utilizes a fluorogenic substrate for activated Caspase-3 and -7. Synthetic siRNAs corresponding to the human *CDCP1* mRNA coding region or *Alu* sense siRNAs (S3–S4) (Supplemental Table S1) were used for knockdown of the *CDCP1* gene. Staurosporine (Cell Signaling Technology) treatment at 2 µM for 2 h was used as positive control. DNA was counterstained with Hoechst 33342 (Life Technologies). Apoptosis levels were also determined by western blotting analysis using cleaved PARP (Asp214) antibody (Supplemental Table S2).

### Statistical analysis

Image quantification was performed with ImageJ software. Data are presented as means ± SD. Two-tailed *t*-tests were conducted, and the minimum level for significance was *P* < 0.05.

## DATA DEPOSITION

Dicing product sequencing data has been deposited in the Sequence Read Archive under series accession number SRR10824153.

## SUPPLEMENTAL MATERIAL

Supplemental material is available for this article.

## Supplementary Material

Supplemental Material
