# Supplementary material for: Processing of Alu small RNAs by DICER/ADAR1 complexes and their RNAi targets
Source: RNA. 2020 Dec;26(12):1801–14. doi: 10.1261/rna.076745.120 (PMC7668262; doi:10.1261/rna.076745.120)
Supplement: Supplemental Material [file supp_26_12_1801__index.html]

Processing of Alu small RNAs by DICER/ADAR1 complexes and their RNAi targets — Supplemental Material 

# Processing of *Alu* small RNAs by DICER/ADAR1 complexes and their RNAi targets

## Supplemental Material

- Supplemental\_Material.pdf
- Supplemental\_TableS1\_.xlsx
- Supplemental\_TableS2\_.xlsx
- Supplemental\_TableS3\_.xlsx
- Supplemental\_TableS4\_.xlsx
