# Supplementary material for: Processing of Alu small RNAs by DICER/ADAR1 complexes and their RNAi targets
Source: RNA. 2020 Dec;26(12):1801–14. doi: 10.1261/rna.076745.120 (PMC7668262; doi:10.1261/rna.076745.120)
Supplement: Supplemental Material [file supp_076745.120_Supplemental_Material.pdf]

SUPPLEMENTAL MATERIAL for

Processing of *Alu* small RNAs by DICER/ADAR1 complexes and their RNAi targets

Yusuke Shiromoto, Masayuki Sakurai, Helen Qu, Andrew V Kossenkov, and Kazuko Nishikura

- Supplemental Figure 1-4
- Supplemental Figure Legends
- Supplemental Table 1- 4

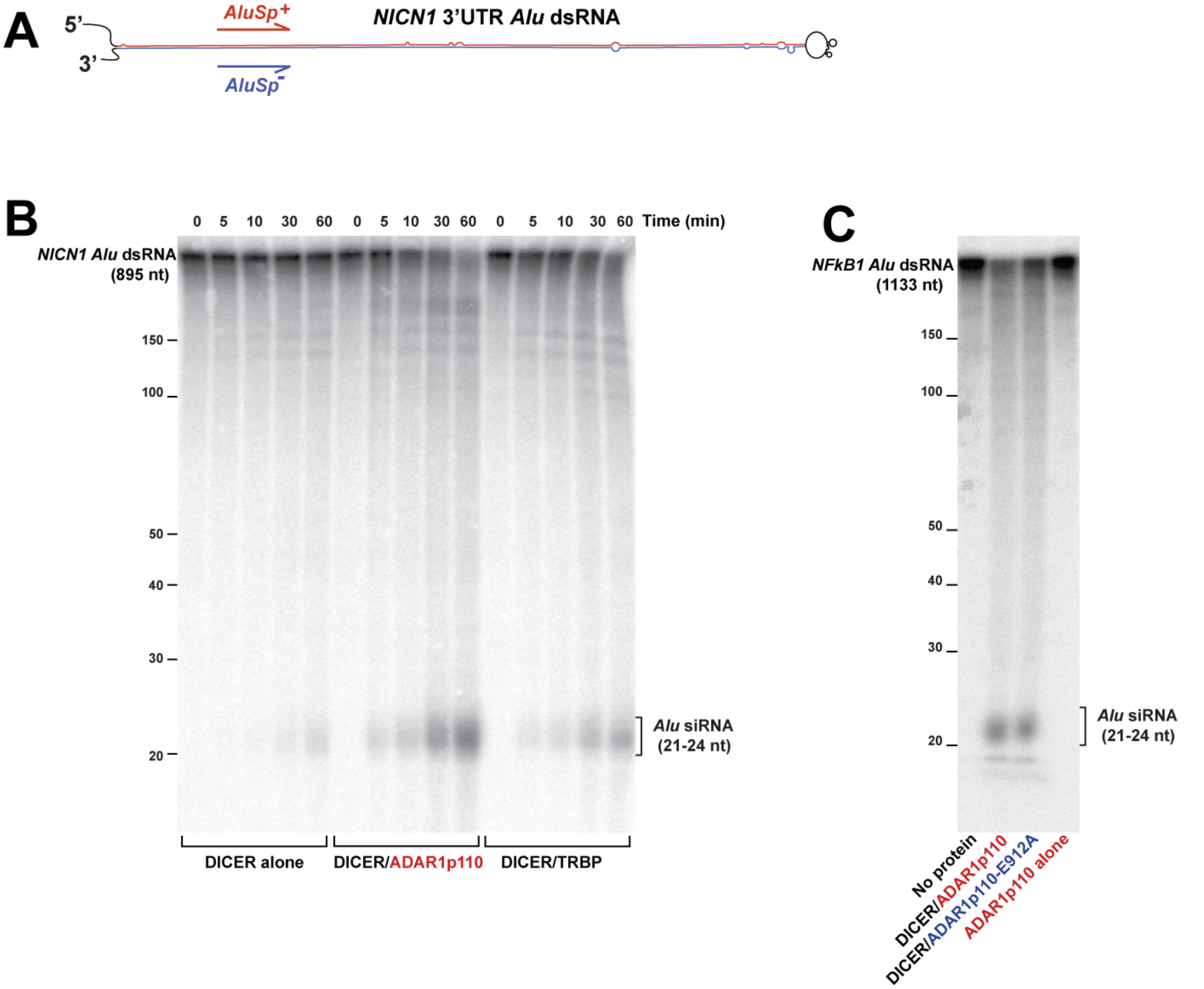

SUPPLEMENTAL FIGURE S1. ADAR1 augments the DICER cleavage reaction rate for *Alu* dsRNAs, and its DICER promoting function does not require its deaminase activity. (*A*) Secondary structure of *NICN1* 3'UTR *Alu* dsRNA. (*B*) The time course analysis for DICER cleavage of *NICN1 Alu* dsRNAs. The DICER reaction was done at 37 °C with 0.15 nM of *NICN1 Alu* dsRNA and 1.5 nM of DICER alone or DICER complexes for various times. (*C*) The DICER cleavage reaction for *NFkB1 Alu* dsRNAs was conducted at 37 °C for 30 min with DICER/ADAR1p110-wild type complex, DICER/ADAR1p110-E912A complex, or ADAR1p110 alone.

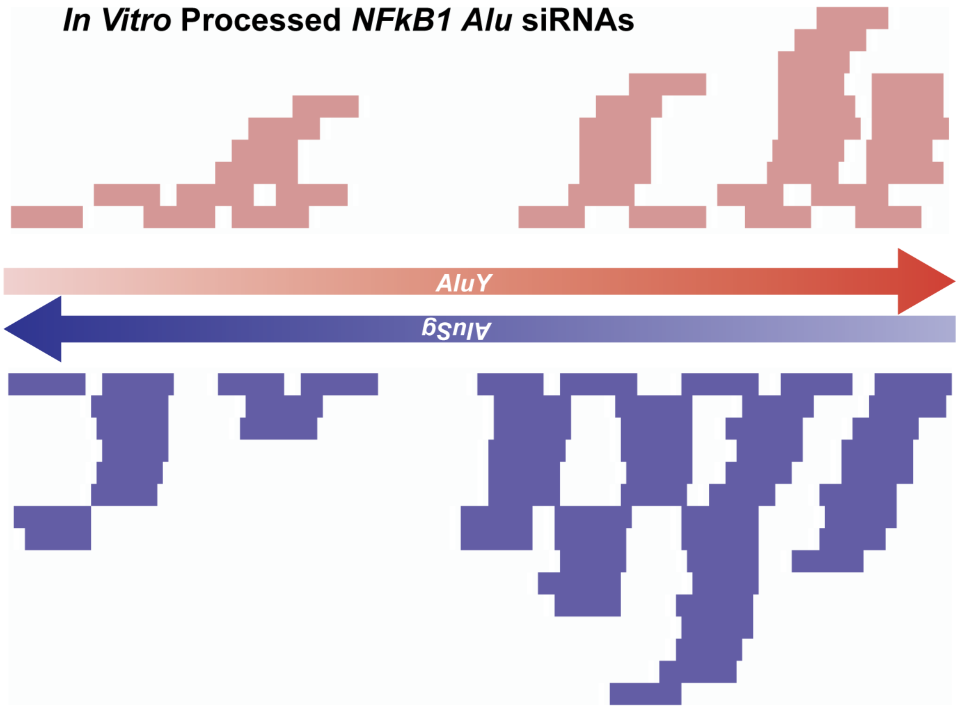

SUPPLEMENTAL FIGURE S2. Distribution of *NFκB1 Alu* siRNAs cleaved *in vitro* by the DICER/ADAR1p110 complex to *NFκB1* intronic *AluY* and *AluSg* sequences. The DICER/ADAR1p110 complex does not have A-to-I editing activity (Ota et al. 2013), and no A-to-G changes were detected in *Alu* siRNA read sequences.

A

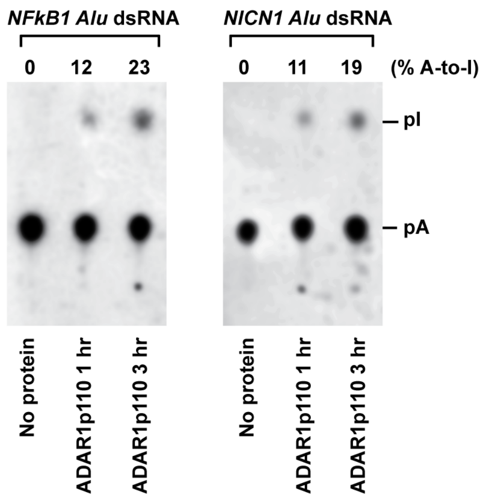

NFkB1 Alu dsRNA
NICN1 Alu dsRNA
0 12 23
0 11 19
(% A-to-I)
pI
pA
No protein
ADAR1p110 1 hr
ADAR1p110 3 hr
No protein
ADAR1p110 1 hr
ADAR1p110 3 hr

B

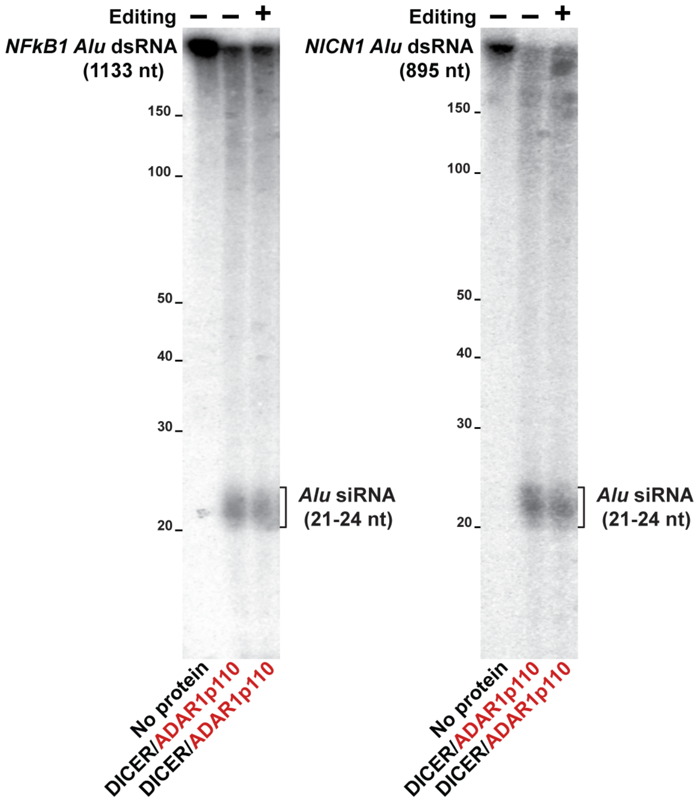

Editing – – +
NFkB1 Alu dsRNA (1133 nt)
150
100
50
40
30
20
Alu siRNA (21-24 nt)
No protein
DICER/ADAR1p110
DICER/ADAR1p110
Editing – – +
NICN1 Alu dsRNA (895 nt)
150
100
50
40
30
20
Alu siRNA (21-24 nt)
No protein
DICER/ADAR1p110
DICER/ADAR1p110

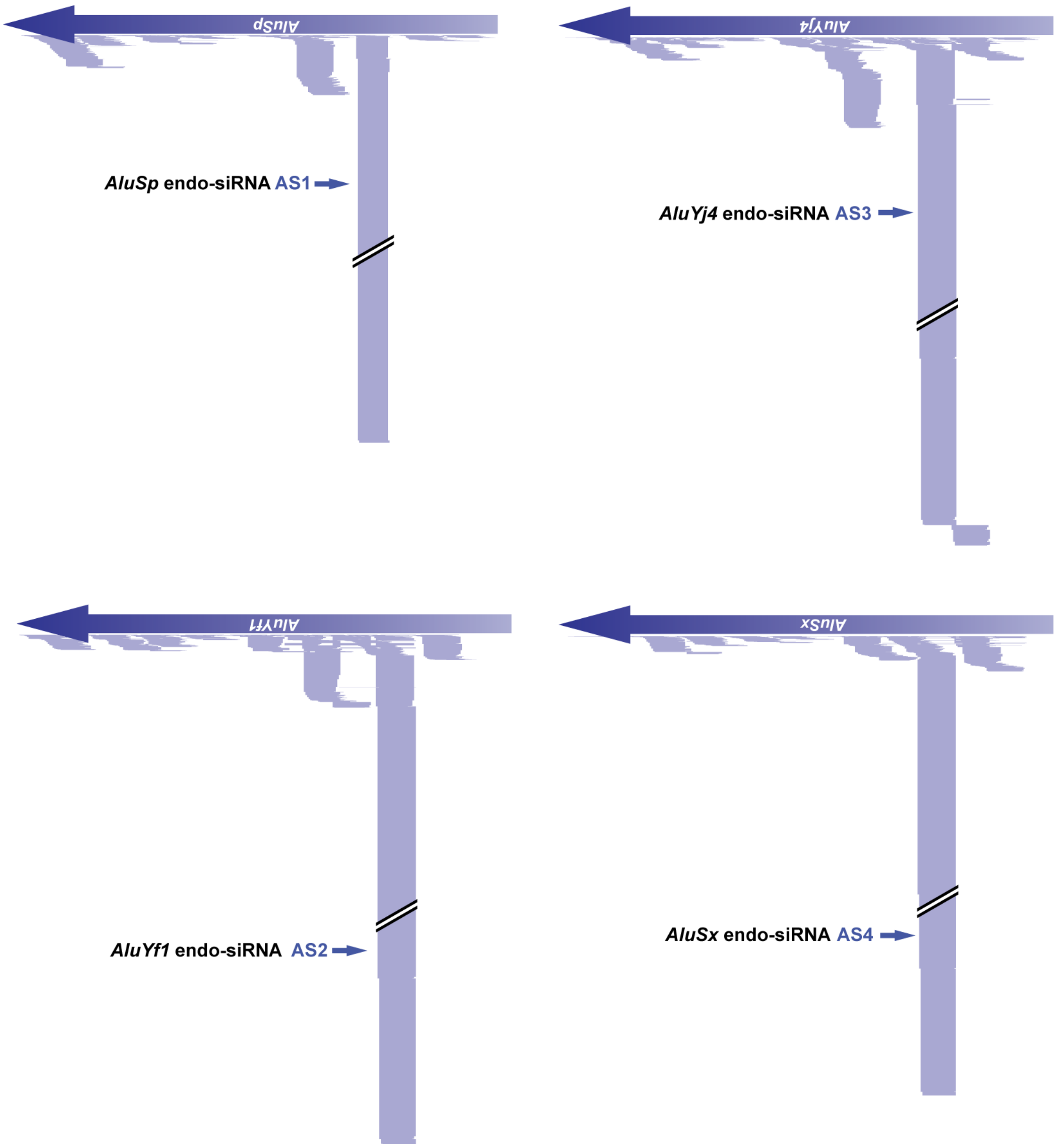

SUPPLEMENTAL FIGURE S4. Antisense *Alu* siRNAs are loaded onto AGO2. Distribution of AGO2-bound *Alu* siRNAs corresponding to antisense strands of *AluSp* (chr1:8801271-8801568), *AluYf1* (chr1:237631614-237631932), *AluYj4* (chr1:172387376-172387672), and *AluSx* (chr1:6777154-6777465).

SUPPLEMENTAL FIGURE LEGENDS

SUPPLEMENTAL FIGURE S1. ADAR1 augments the DICER cleavage reaction rate for *Alu* dsRNAs, and its DICER promoting function does not require its deaminase activity. (*A*) Secondary structure of *NICN1* 3'UTR *Alu* dsRNA. (*B*) The time course analysis for DICER cleavage of *NICN1 Alu* dsRNAs. The DICER reaction was done at 37 ^{o}C with 0.15 nM of *NICN1 Alu* dsRNA and 1.5 nM of DICER alone or DICER complexes for various times. (*C*) The DICER cleavage reaction for *NFkB1 Alu* dsRNAs was conducted at 37 ^{o}C for 30 min with DICER/ADAR1p110-wild type complex, DICER/ADAR1p110-E912A complex, or ADAR1p110 alone.

SUPPLEMENTAL FIGURE S2. Distribution of *NFκB1 Alu* siRNAs cleaved *in vitro* by the DICER/ADAR1p110 complex to *NFκB1* intronic *AluY* and *AluSg* sequences. The DICER/ADAR1p110 complex does not have A-to-I editing activity (Ota et al. 2013), and no A-to-G changes were detected in in *Alu* siRNA read sequences.

SUPPLEMENTAL FIGURE S3. A-to-I editing of *Alu* dsRNA does not affect its cleavage efficiency by DICER/ADAR1p110 complex. (*A*) *In vitro* A-to-I editing assay was carried out using ADAR1p110 recombinant protein and ^{32}P-ATP labeled *NFκB1 Alu* dsRNA or *NICN1 Alu* dsRNA. A-to-I conversion was quantitated using TLC assay. A-to-I editing of the dsRNA stem region was estimated as 54% for *NFκB1 Alu* dsRNA and 34% for *NICN1 Alu* dsRNA. (*B*) The DICER reaction was done at 37 ^{o}C for 30 min with 1.5 nM of DICER/ADAR1p110 complex and 0.15 nM of *NFκB1 Alu* dsRNA or *NICN1 Alu* dsRNA edited to maximum levels for 3 hrs.

SUPPLEMENTAL FIGURE S4. Antisense *Alu* siRNAs are loaded onto AGO2. Distribution of AGO2-bound *Alu* siRNAs corresponding to antisense strands of *AluSp* (chr1:8801271-8801568), *AluYf1* (chr1:237631614-237631932), *AluYj4* (chr1:172387376-172387672), and *AluSx* (chr1:6777154-6777465).

SUPPLEMENTAL REFERENCES

Ota H, Sakurai M, Gupta R, Valente L, Wulff BE, Ariyoshi K, Iizasa H, Davuluri RV, Nishikura K. 2013. ADAR1 forms a complex with Dicer to promote microRNA processing and RNA-induced gene silencing. *Cell* **153**: 575-589.

SUPPLEMENTAL TABLE LEGENDS

SUPPLEMENTAL TABLE 1. DNA and RNA oligos used in this study.

SUPPLEMENTAL TABLE 2. Antibodies used in this study.

SUPPLEMENTAL TABLE 3. AGO2 bound *Alu* endo-siRNAs.
The sequences and read numbers of AGO2 bound *Alu* siRNAs are listed.

SUPPLEMENTAL TABLE 4. Genes containing a single *Alu* sequence within their 3'UTRs. (*A*) Genes containing an antisense *Alu* sequence within their 3'UTRs. (*B*) Genes containing a sense *Alu* sequence within their 3'UTRs.
